# Substituting Doxorubicin with Nonpegylated Liposomal Doxorubicin for the Treatment of Early Breast Cancer: Results of a Retrospective Study

**DOI:** 10.1155/2014/984067

**Published:** 2014-01-12

**Authors:** Neville Davidson, Teresa Camburn, Ian Keary, David Houghton

**Affiliations:** ^1^Broomfield Hospital, Court Road, Chelmsford, Essex CM1 7ET, UK; ^2^Rivers Hospital, High Wych Road, Sawbridgeworth Hertfordshire CM21 0HH, UK; ^3^Strategen Limited, 2 & 3 Stable Court, Herriard Park Estate, Herriard, Basingstoke RG25 2PL, UK

## Abstract

*Introduction*. Evidence from the metastatic setting suggests that replacing conventional doxorubicin with nonpegylated liposomal doxorubicin (NPLD) for early breast cancer may maintain efficacy whilst reducing long-term cardiotoxicity, an important consideration with many patients going on to receive multiple lines of treatment. *Methods*. Consecutive patients with early breast cancer treated with NPLD were assessed for disease progression and changes in cardiac function according to left ventricular ejection fraction (LVEF). *Results*. Ninety-seven patients (median age at diagnosis 51 (32–76) years) were studied. The majority received NPLD (60 mg/m^2^ plus cyclophosphamide 600 mg/m^2^) adjuvantly (79.4%) and in sequence with a taxane (79.4%; docetaxel 75 mg/m^2^). 80.4% had radiotherapy and 15.5% received trastuzumab. Mean time to disease recurrence was 87.0 months (80.7–93.2 [95% confidence interval]) and 5-year disease-free survival was 86.0%. Mean LVEF values remained within the normal range of ≥55% during treatment and throughout the cardiac follow-up period (median 7 months, range 1–21 months). Use of trastuzumab and age at diagnosis did not appear to influence LVEF. *Conclusion*. NPLD appeared to be a well-tolerated substitute for conventional doxorubicin in patients with early breast cancer.

## 1. Introduction

Nonpegylated liposomal doxorubicin (NPLD; Myocet, Teva UK) has potential advantages over conventional doxorubicin in the treatment of early breast cancer. Utilising a less cardiotoxic but equally effective treatment earlier in management may help to maximise therapeutic options later in the course of disease and thereby facilitate the use of multiple lines of therapy. In addition, substituting NPLD for doxorubicin as the standard anthracycline in early breast cancer may help address the growing concerns regarding the longer-term impact of treatment on cardiac function, a key survivorship issue [1, 2]. However, whilst NPLD has been extensively studied in metastatic breast cancer and is licensed in this regard [3–7], data on its use in early disease are currently scarce [8, 9].

As advances in diagnosis, management, and treatment have led to improved breast cancer survival, the issue of longer-term, therapy-related cardiotoxicity has taken on increasing importance, with patients potentially facing multiple, coincident insults to the heart [10]. Many of the available adjuvant therapies, which are increasingly used in combination or sequence, have been associated with some form of cardiotoxicity during or after therapy, whilst increasing age, comorbid conditions, and disease-related decreases in physical activity can also contribute to cardiovascular disease [10]. In addition to cumulative cardiotoxicity potentially limiting treatment options and therefore outcomes, acute improvements in breast cancer survival may be offset by increases in cardiovascular disease and mortality [1, 2]. Therefore, the challenge for the clinician, particularly in the early breast cancer setting, is to balance the need for anticancer efficacy against the potential for subsequent treatment-related cardiovascular disease and mortality.

Data from the metastatic setting, which demonstrate that NPLD is equally efficacious but significantly less cardiotoxic than conventional doxorubicin [3, 4, 6], provide a good rationale to postulate that NPLD may help achieve this delicate balance in the adjuvant setting. The results of two small phase II studies [8, 9] suggest that the benefits observed in metastatic patients may be maintained in early breast cancer, but further data are required. The objective of our study, therefore, was to assess the use of NPLD in place of conventional doxorubicin in standard regimens as a novel therapeutic option for the treatment of patients with early breast cancer.

## 2. Materials and Methods

The objective of this study was to assess the efficacy and cardiac tolerability of NPLD as a substitute for conventional doxorubicin in patients with early breast cancer in routine clinical practice. Efficacy was assessed in terms of disease recurrence and survival, whilst cardiac function was assessed according to left ventricular ejection fraction (LVEF).

The medical records of all patients with early breast cancer receiving treatment with NPLD at the Rivers Hospital, Chelmsford, UK, between January 2003 and August 2011 were retrospectively reviewed and the following information was extracted: date of diagnosis, date of surgery, TNM stage, type of surgery, chemotherapy regimen, radiotherapy schedule, use of endocrine therapy, use of trastuzumab, and the dates of LVEFs derived from any echocardiograms. Details of disease recurrence and mortality were also recorded. All data were anonymised. Ethical review and patient consent were deemed unnecessary by the local review board, in line with the National Research Ethics Service (NRES) guidance document, as the study was considered a service evaluation [11].

### 2.1. Data Analysis

Time to recurrence and 5-year disease-free survival were estimated for the total study population using Kaplan-Meier analysis. All patients who had the minimum of a baseline (i.e., immediately prior to or during the first cycle of chemotherapy) echocardiogram were included in the analysis of cardiac function. To facilitate analysis, echocardiogram results were grouped according to when they were taken in relation to the timing of chemotherapy. If a patient had more than one reading in a given time period, the lowest LVEF obtained was used. Additional analyses were performed for the following subgroups: primary versus adjuvant therapy, NPLD regimen used, whether patients received adjuvant trastuzumab or not, and patient age (≥60 years versus <60 years). Patients with LVEF values ≥55% were considered to have normal cardiac function, as defined by current British Society of Echocardiography guidelines [10].

## 3. Results

A total of 97 women (median age at diagnosis 51 [32–76] years) were studied (Table 1). Seventy-seven (79.4%) patients were treated adjuvantly, with the remainder receiving NPLD as primary therapy. NPLD was administered at a dosage of 60 mg/m^2^ combined with cyclophosphamide (600 mg/m^2^) for 4 or 6 cycles. The majority (79.4%) of patients received NPLD in sequence with a taxane (docetaxel 75 mg/m^2^). In total, 15.5% of patients received trastuzumab and 80.4% had radiotherapy. The median followup from diagnosis was 48 months (range 9–114 months).

### 3.1. Disease Recurrence or Progression

**Figure 1:**
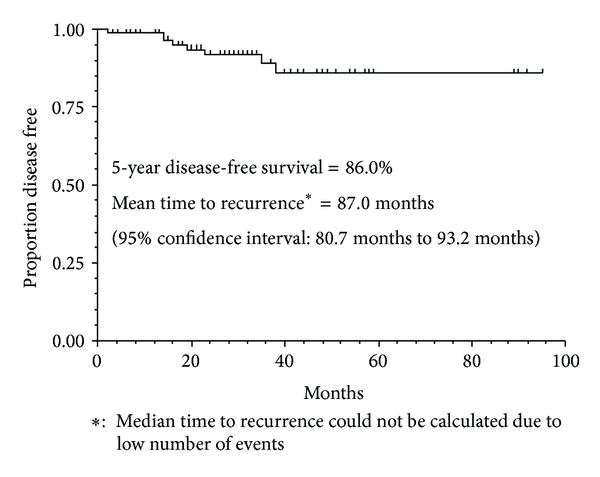
Kaplan-Meier survival plot for disease-free survival following surgery (*n* = 97).

Eight of the 97 (8.2%) patients experienced disease recurrence or progression and 2 (2.1%) died during the study period. Of the 8 patients who relapsed/progressed, 5 had received AC-T without trastuzumab, 2 had received AC-T with trastuzumab, and 1 had received TAC without trastuzumab. The sites of recurrence included bone, lymph nodes, liver, lung, and the contralateral breast. Kaplan-Meier analysis estimated that the mean time to recurrence was 87.0 months after surgery and that 5-year disease-free survival was 86.0% (Figure 1).

### 3.2. Cardiac Function

**Figure 2:**
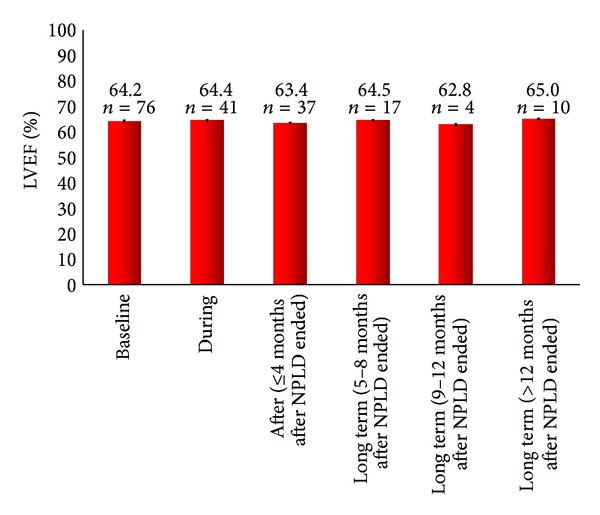
Mean (± standard error) LVEF values in the overall patient population.

Seventy-six patients had at least a baseline echocardiogram and were included in the cardiac function analysis. The median cardiac follow-up period (i.e., time between the baseline and last recorded echocardiogram) was 7 months (range 1–21 months). Mean LVEF values remained within the normal range (≥55%) and were stable at baseline (64.2%), during chemotherapy (64.4%), and at other specified time points over the study period (range: 62.8%–65.0%; Figure 2). In total, 9 patients had at least one LVEF in the range 45%–54%, which corresponds to a mild depression of LVEF [10]. Of these patients, 4 had baseline LVEFs of 45%–54% that returned to the normal range following chemotherapy, 3 experienced a fall in LVEF to 45%–54% during chemotherapy but returned to normal levels following treatment completion, 1 patient had a fall of 13 points from 65% at baseline to 52% during therapy, and 1 patient had a fall of 6 points from 58% to 52% during therapy; no further data for either patient were available beyond this time.

Similarly, no differences in mean LVEF values during or after chemotherapy were observed when patients were compared on the basis of timing of NPLD (primary versus adjuvant), NPLD regimen (AC versus TAC. versus AC + T) or patient age (<60 years versus ≥60 years) (Table 2).

**Figure 3:**
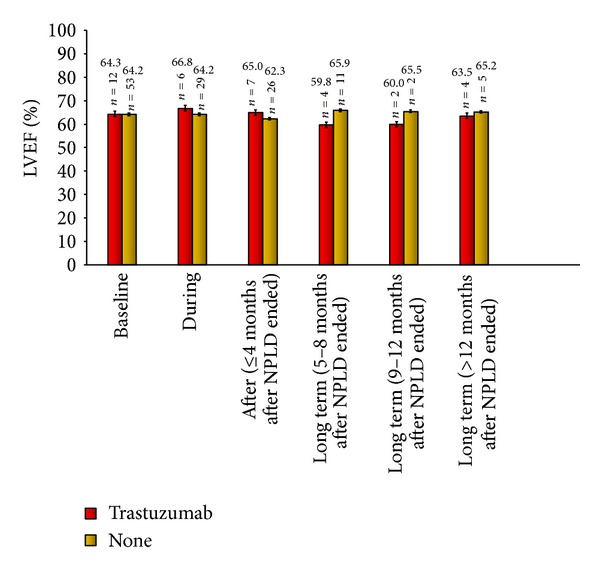
Mean (± standard error) LVEF values in patients who did or did not receive trastuzumab.

No differences in the mean LVEF value were apparent at any time point among those who did and did not receive trastuzumab. Mean LVEF remained in the normal range at baseline (64.3% versus 64.2%, resp.), during chemotherapy (66.8% versus 64.2%) and at other specified time points during the study period (Figure 3). Limiting the analysis to those patients who received NPLD and trastuzumab as adjuvant therapy did not change the outcome (data not shown).

## 4. Discussion

The results of our study suggest that NPLD offers an attractive alternative to conventional doxorubicin in the treatment of early breast cancer. When used in standard primary and adjuvant regimens, NLPD appeared to have no detrimental effect on cardiac function. Five-year disease-free survival was 86%; however, interpretation concerning efficacy is constrained by the small size of the study. It is interesting to speculate whether with greater followup, the apparently reduced cardiotoxicity of NPLD would be reflected in improvements in survival through decreased cardiac mortality.

Cardiac function, as assessed by LVEF measurements, remained clinically stable throughout the follow-up period regardless of NPLD timing or regimen, patient age, or trastuzumab use. Indeed, mean LVEF values did not fall below the British Society of Echocardiography [10] definition of a normal LVEF of 55% in any of the analyses carried out. Individually, 9 patients had LVEFs of 45%–55% recorded; however, this appeared to be reversible, and, to date, none of the patients included in this study have represented with cardiac-related problems.

Our finding that the mean LVEF remained comparable in patients who did and did not receive trastuzumab may have important implications for future treatment strategies. It is well-documented that the use of trastuzumab in combination with anthracyclines has potent antitumour potential but at the cost of prohibitively increased cardiotoxicity [12, 13]. Our results suggest that use of NPLD should be further investigated both as an alternative to conventional anthracyclines, to maximise subsequent therapy options and thereby as a means of minimising cardiotoxicity-related interruption of trastuzumab therapy, and also as a combined regimen with trastuzumab. Indeed, use of these two agents in combination is already an area of increasing interest [14–16], with preliminary results to date suggesting that it might be a favourable therapeutic approach [15, 16].

We recognise that our study is limited by its single centre, retrospective design, lack of an appropriate comparator group, and limited follow-up period in terms of the number of patients with recorded LVEF measurements following the completion of chemotherapy. However, the consistency of our results is encouraging, and we believe that they demonstrate that NPLD may offer a basis for novel treatment regimens for early breast cancer with improved therapeutic indices compared to current standards of care. Further data are required to confirm these promising findings, particularly in the longer term.

## 5. Conclusions

Nonpegylated liposomal doxorubicin (NPLD; Myocet) has been shown to improve the therapeutic index of conventional doxorubicin by significantly reducing the risk of cardiotoxicity when used as first-line therapy for metastatic breast cancer [3, 4, 15, 16]. Utilising a less cardiotoxic but equally effective treatment earlier in the management of breast cancer may help to maximise therapeutic options later in the course of disease and thereby facilitate the use of multiple lines of therapy. In addition, substituting NPLD for conventional doxorubicin as the standard anthracycline in early breast cancer (EBC) may help address the growing concerns regarding the longer-term impact of treatment on cardiac function [1, 2]. Current evidence on the use of NPLD in early disease is restricted to two phase II trials [8, 9], both of which report limited cardiac data for NPLD.

In our study, NPLD did not have a detrimental effect on cardiac function, which was maintained for >12 months after the completion of treatment. Indeed, mean LVEF values did not fall below the British Society of Echocardiography [10] definition of a normal LVEF of 55% in any of the analyses carried out. Furthermore, the addition of trastuzumab to NPLD regimens did not result in increased cardiotoxicity. These findings suggest that NPLD may offer a basis for novel treatment regimens for EBC with decreased long-term cardiac impact compared to current standards of care.

## Figures and Tables

**Table 1:** Baseline demographic characteristics.

Number of evaluable patients	97
Median age at diagnosis (range)	51 (32–76)
TNM stage [*n* (%)]	
I	20 (20.6%)
II	41 (42.3%)
IIA	4 (4.1%)
IIB	5 (5.2%)
III	21 (21.6%)
IIIA	5 (5.2%)
Unknown	1 (1.0%)
Type of surgery [*n* (%)]	
Mastectomy	51 (52.6%)
Wide local excision	46 (47.4%)
Chemotherapy regimen [*n* (%)]	
Primary	20 (20.4%)
Adjuvant	78 (79.6%)
Anthracycline regimen [*n* (%)]*	
AC	4 (4.1%)
AC + T	77 (79.4%)
TAC	16 (16.5%)
Trastuzumab [*n* (%)]	
Yes	15 (15.5 %)
No	70 (72.2%)
Unknown	12 (12.4%)
Radiotherapy [*n* (%)]	
Yes	78 (80.4%)
No	19 (19.6%)
Endocrine therapy	
Yes	72 (74.2%)
No	5 (5.2%)
Unknown	20 (20.6%)

*AC: 6 cycles of NPLD (60 mg/m^2^) and cyclophosphamide (600 mg/m^2^).

AC + T: 4 cycles of NPLD (60 mg/m^2^) and cyclophosphamide (600 mg/m^2^) followed by 4 cycles of docetaxel (75 mg/m^2^).

TAC: six cycles of NPLD (60 mg/m^2^) and cyclophosphamide (600 mg/m^2^) and docetaxel (75 mg/m^2^).

**Table 2:** Mean (±standard error) LVEF (%) values according to timing of NPLD, regimen used, and age at diagnosis.

		Baseline	During	≤4 months after chemotherapy ended	5–8 months after chemotherapy ended	9–12 months after chemotherapy ended	>12 months after chemotherapy ended
NPLD timing	Adjuvant	63.9 ± 0.7 (*n* = 60)	64.8 ± 0.8 (*n* = 33)	63.4 ± 1.3 (*n* = 31)	64.8 ± 1.7 (*n* = 11)	62.8 ± 1.6 (*n* = 4)	64.1 ± 1.6 (*n* = 8)
	Primary	65.1 ± 1.3 (*n* = 17)	63.0 ± 1.6 (*n* = 8)	63.3 ± 1.5 (*n* = 6)	63.8 ± 2.8 (*n* = 6)	No data	68.5 ± 1.5 (*n* = 2)

NPLD regimen	AC + T	63.8 ± 0.7 (*n* = 59)	63.9 ± 0.8 (*n* = 27)	63.3 ± 1.3 (*n* = 29)	64.7 ± 1.5 (*n* = 12)	63.7 ± 1.9 (*n* = 3)	64.6 ± 1.7 (*n* = 7)
	TAC	66.1 ± 1.5 (*n* = 14)	65.4 ± 1.7 (*n* = 12)	63.9 ± 2.0 (*n* = 7)	64.0 ± 3.6 (*n* = 5)	60.0 (*n* = 1)	66.0 ± 3.1 (*n* = 3)
	AC	62.7 ± 2.7 (*n* = 3)	66.0 ± 2.0 (*n* = 2)	60.0 (*n* = 1)	No data	No data	No data

Age at diagnosis	<60	64.0 ± 0.8 (*n* = 53)	65.1 ± 0.8 (*n* = 29)	62.9 ± 1.5 (*n* = 25)	64.0 ± 1.6 (*n* = 15)	62.8 ± 1.6 (*n* = 4)	64.9 ± 1.6 (*n* = 8)
	≥60	64.5 ± 1.0 (*n* = 23)	62.8 ± 1.6 (*n* = 12)	64.3 ± 1.4 (*n* = 12)	68.0 ± 2.0 (*n* = 2)	No data	64.5 ± 2.5 (*n* = 2)

AC: 6 cycles of NPLD (60 mg/m^2^) and cyclophosphamide (600 mg/m^2^).

AC + T: 4 cycles of NPLD (60 mg/m^2^) and cyclophosphamide (600 mg/m^2^) followed by 4 cycles of docetaxel (75 mg/m^2^).

TAC: six cycles of NPLD (60 mg/m^2^) and cyclophosphamide (600 mg/m^2^) and docetaxel (75 mg/m^2^).

## Abbreviations

NPLD: Nonpegylated liposomal doxorubicin
LVEF: Left ventricular ejection fraction
TNM: Tumour node metastasis
AC: Anthracycline
AC-T: Sequential anthracycline then taxane
TAC: Combination taxane and anthracycline
EBC: Early Breast Cancer
NRES: National Research Ethics Service.
